# Effects of multicomponent exercise on cognitive function in older adults with amnestic mild cognitive impairment: a randomized controlled trial

**DOI:** 10.1186/1471-2377-12-128

**Published:** 2012-10-31

**Authors:** Takao Suzuki, Hiroyuki Shimada, Hyuma Makizako, Takehiko Doi, Daisuke Yoshida, Kota Tsutsumimoto, Yuya Anan, Kazuki Uemura, Sangyoon Lee, Hyuntae Park

**Affiliations:** 1 Research Institute, National Center for Geriatrics and Gerontology, Obu, Japan; 2 Section for Health Promotion, Department for Research and Development to Support Independent Life of Elderly, Center for Gerontology and Social Science, National Center for Geriatrics and Gerontology, Obu, Japan; 3 Section for Physical Functioning Activation, Department of Functioning Activation, Center for Gerontology and Social Science, National Center for Geriatrics and Gerontology, Obu, Japan

**Keywords:** Aerobic exercise, MCI, Elderly, Alzheimer’s disease, Prevention

## Abstract

**Background:**

To examine the effects of a multicomponent exercise program on the cognitive function of older adults with amnestic mild cognitive impairment (aMCI).

**Methods:**

Design: Twelve months, randomized controlled trial; Setting: Community center in Japan; Participants: Fifty older adults (27 men) with aMCI ranging in age from 65 to 93 years (mean age, 75 years); Intervention: Subjects were randomized into either a multicomponent exercise (n = 25) or an education control group (n = 25). Subjects in the multicomponent exercise group exercised under the supervision of physiotherapists for 90 min/d, 2 d/wk, for a total of 80 times over 12 months. The exercises included aerobic exercises, muscle strength training, and postural balance retraining, and were conducted using multiple conditions to stimulate cognitive functions. Subjects in the control group attended three education classes regarding health during the 12-month period. Measurements were administered before, after the 6-month, and after the 12-month intervention period; Measurements: The performance measures included the mini-mental state examination, logical memory subtest of the Wechsler memory scale-revised, digit symbol coding test, letter and categorical verbal fluency test, and the Stroop color word test.

**Results:**

The mean adherence to the exercise program was 79.2%. Improvements of cognitive function following multicomponent exercise were superior at treatment end (group × time interactions for the mini-mental state examination (*P* = 0.04), logical memory of immediate recall (*P* = 0.03), and letter verbal fluency test (*P* = 0.02)). The logical memory of delayed recall, digit symbol coding, and Stroop color word test showed main effects of time, although there were no group × time interactions.

**Conclusions:**

This study indicates that exercise improves or supports, at least partly, cognitive performance in older adults with aMCI.

## Background

Population-based studies in older adults performed in North America, Europe, and Asia report a prevalence of mild cognitive impairment (MCI) ranging from 11% to 17% [1-5], and a prevalence of the amnestic MCI (aMCI) subtype between 3 and 5% [2,6]. Evidence from both neuropsychological and neuroimaging studies have suggested that MCI represents a clinical prodrome to degenerative dementias such as Alzheimer’s dementia (AD) [7,8]. This is particularly the case with aMCI, which is likely to progress to AD [9]. Early treatment of mild to moderate AD is associated with better responses than later treatment [10], so it is conceivable that treating MCI may be particularly effective in delaying the progression to AD.

Clinical aspects have been widely examined as possible biomarkers for MCI to detect subjects at greater risk of conversion to dementia. Neuropsychological predictors of conversion include performance on specific cognitive tests, particularly those assessing delayed recall and executive functions [11-14]. Reducing or recovering from identified risk factors is important in the prevention of the conversion from MCI to AD or to delay the progression of the prodromal symptoms of dementia.

Epidemiological data suggests that moderate exercise and physical activity, such as walking, are associated with a lower risk of dementia [15]. According to these findings, epidemiological studies and randomized controlled trials examining the effects of exercise have proposed it is associated with various cognitive benefits [16-25], and a meta-analysis reported that physical activity or exercise is associated with improvements in attention, processing speed, and executive function in older adults with or without cognitive impairments [26-28]. RCT have been conducted to determine the effects of exercise or physical activity on cognitive functions in older adults with MCI. These studies identified the effects of exercise or physical activity on cognitive function including general cognitive function, executive function and glucometabolic and hypothalamic-pituitary-adrenal axis responses in older adults with MCI [16,19,22,23]. However, because the results of these studies differed largely due to differences in methodology, sufficient evidence has not been garnered regarding the relationship between exercise and cognitive function in aMCI.

We designed the present randomized trial to test whether 12 months of supervised multicomponent exercise improved cognitive function among older adults with aMCI. The multicomponent exercise included aerobic exercise, muscle strength training, and postural balance retraining. We adopted the multicomponent regimen because a previous review suggested that participants in combined aerobic and strength training regimens improved cognitive function to a reliably greater degree than those in aerobic training alone (0.59 vs. 0.41, SE = 0.043, n = 101, p < 0.05) [29].

## Methods

### Participants

In this 12-month randomized controlled trial, the subjects were divided into a multicomponent exercise or an education control group at the end of the baseline assessment. Study personnel involved in collection of outcome measures were unaware of each subject’s assigned group. The Ethics Committee of the National Center for Geriatrics and Gerontology approved the study protocol. The purpose, nature, and potential risks of the experiments were fully explained to the subjects, and all subjects gave written, informed consent before participating in the study.

**Figure 1:**
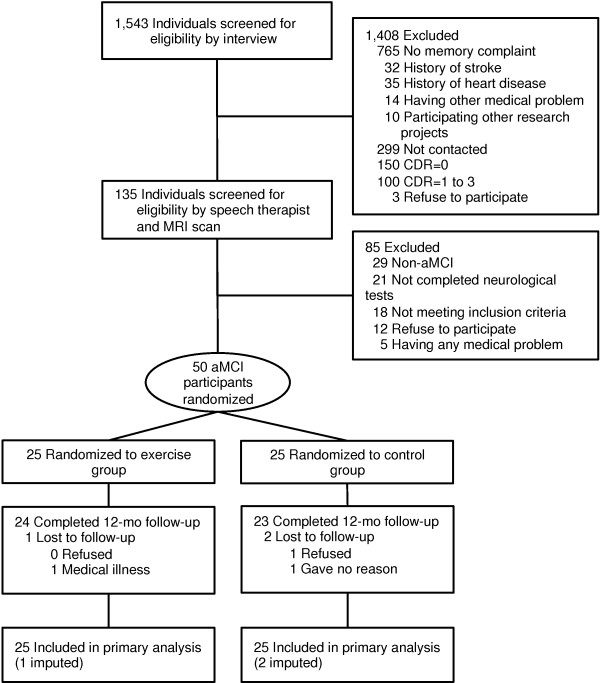
Subject flow diagram from the initial contact through the study completion.

Subjects in this study were recruited from our volunteer databases, which included elderly individuals (65 years and over). The inclusion criteria we used were dwelling in the community and being 65 years or age or older. 528 prospective subjects with a Clinical Dementia Rating (CDR) [30] of 0.5, or who complained of memory impairments, were recruited for the initial eligibility assessments. 135 subjects participated in the secondary eligibility assessments, including neuropsychological tests and magnetic resonance imaging. 35 of these 135 subjects were excluded and the remaining 100 subjects met the definition of MCI using the Petersen criteria [31]. The objective memory impairment was defined as having a lower memory in the Logical Memory II subtest of the Wechsler memory scale-revised (WMS-LM II) [32]. The cut-off score to define the aMCI was adjusted by educational history (0–9 years: <7 points, 10–15 years: <10 points, more than 16 years: <12 points) [33]. The exclusion criteria we used included having a CDR = 0, 1, 2, and 3, a history of neurological, psychiatric, and cardiac disorders or other severe health issues, use of donepezil, loss of independence in basic activities of daily living (ADL), and current participation in other research projects. A diagram consistent with the Consolidated Standards of Reporting Trials (CONSORT) [34] that outlines the subject flow from first contact to study completion is shown in Figure 1. Fifty older adults (mean age 76.0 ± 7.1 years, range 65–92 years, male = 27 (54 %), mean education level 10.9 ± 2.5 years) with aMCI who had objective memory disabilities and who completed the neuroimaging assessments were selected as the subjects in the study.

### Intervention

Subjects in the multicomponent exercise group exercised under the supervision of physiotherapists for 90 min/d, 2 d/wk, for a total of 80 times over 12 months. Two physiotherapists involved in geriatric rehabilitation and three well-trained instructors conducted each intervention. The exercise class consisted of 16–17 participants, and each supervised session began with a 10-min warm-up period, followed by 20 min of muscle strength exercise. The subjects then practiced aerobic exercise, postural balance retraining, and dual-task training for 60 min. In the aerobic exercises and postural balance retraining, subjects completed circuit training including stair stepping, endurance walking, and walking on balance boards. The mean intensity of the aerobic exercises was approximately 60% of maximum heart rate. Heart rate was monitored after aerobic exercise each session to take their pulse. One out of every four classes during the intervention period included outdoor walking during approximately 20–30 minutes. These exercises or training were also conducted under multitask conditions. For example, the subjects in the exercise group were asked to invent their own poem while walking. In the ladder training, subjects memorized a step pattern in consecutive square segments, and were instructed to step as quickly and accurately as possible. Before and after each session of the program, physiotherapists conducted a health check of each subject. The physiotherapists and well-trained instructors provided ongoing safety monitoring to prevent adverse accidents such as falling during the program. Daily home-based exercise in addition to structured program and outdoor walking was recommended to the exercise group. The subjects allocated the exercise group were asked to recording the amount of time spent on daily home-based exercise and the daily total steps for pedometer in a notebook. The subjects made graphs from the records of amount of time and steps to promote active lifestyle through a self-monitoring. Attendance at each session was recorded and a transportation service was provided for the participants, if necessary, to help the subjects maintain their participation in the program.

Subjects in the education control group attended three education classes regarding health promotion during the 12-month study period. The class provided information regarding aging, healthy diet, oral care, brain image diagnosis, prevention of urinary incontinence, and health checks. However, the group did not receive specific information regarding exercise, physical activity, or cognitive health.

### Outcomes

The cognitive function before, after 6-month, and after 12-month intervention period was measured by speech therapists. Prior to the commencement of the study, all staff received training from the authors in the correct protocols for administering all of the assessment measures included in the study. The assessors introduced and demonstrated the assessments to facilitate understanding of the tests by the participants before they conducted the tests. A speech therapist calculated all of the results of the cognitive function tests.

The mini-mental state examination (MMSE) was measured as a general cognitive function [35]. The WMS-LM I and II [32] was used to assess logical memory function. In the WMS-LM, two short stories (story A and B) were read aloud to the subjects, who were then instructed to recall details of the stories immediately (WMS-LM I) and after 30 min (WMS-LM II) (total recall score = 50) [32]. The digit symbol-coding (DSC) subset of the Wechsler Adult Intelligence Scale III was used to assess processing speed [36]. Verbal fluency [37,38] was measured by the number of words generated across 60-second trials [37]. The subject’s listed words began with a letter composed of Japanese characters for the three trials (letter verbal fluency test: LVFT) and that belonged to a semantic category (category verbal fluency test: CVFT). The total number of correct responses was used for the analysis. The Stroop Color and Word Test (SCWT) [39] was used to assess executive function. For the Stroop test, we used two conditions. First, the subjects were instructed to read out words printed in black ink (e.g., *blue*) (SCWT-I). Second, they were shown a page with color words printed in incongruent colored inks (e.g., the word *blue* printed in red ink). The subjects were asked to name the ink color in which the words were printed (while ignoring the word itself) (SCWT-III). There were 24 trials for each condition, and we recorded the time the participants took to read each condition.

### Analysis

The statistical analyses were conducted using SPSS software (Version 20; SPSS Inc., Chicago, IL, USA). The independent samples *t*-test or Chi-square test was used to compare the basic characteristics between the exercise and education control group. The effect of intervention (exercise vs control) over time on the cognitive functions was investigated in intention-to-treat (ITT) analyses. Measurements were analyzed using linear mixed models; analyses assumed missing at random with missingness allowed to be driven by variables included in the analyses. All models included random intercepts to account for correlations between the repeated measures for each participant. The fixed components of the models included effects of group and time and a group × time interaction. To assess the presence of a group and time effect, where this may change over time, we first determined the existence of time, group, and group × time interactions. The post hoc analyses were made between times and groups using the Bonferroni method. All statistical significance tests were two-sided, and an alpha-level of 0.05 was considered statistically significant.

## Results

### Adherence to intervention

There were no significant differences in the baseline characteristics between the exercise and education control groups (Table 1). Figure 1 shows the flow of the participants from the time of screening to study completion at 12 months. 47 (exercise group, n = 24) subjects completed the 12-month follow-up. Two of the twenty-five subjects in the exercise group (1 man and 1 woman) did not attend a single session, but were included in the following analyses. The mean adherence to the exercise program, including these subjects, was 79.2%, and 17 subjects (68.0%) in the exercise group attended our intervention program with more than 80% adherence.

**Table 1:** Baseline characteristics of the subjects

**Characteristic**	**Exercise (n=25)**	**Control (n=25)**
Age, mean ± SD, y	75.3 ± 7.5	76.8 ± 6.8
Men, No. (%), n	13 (52.0)	14 (56.0)
Educational level, mean ± SD, y	11.1 ± 2.4	10.8 ± 2.7
Blood pressure, mean ± SD, mmHg		
Diastolic	77.3 ± 11.1	74.3 ± 10.1
Systolic	152.2 ± 21.0	143.7 ± 21.3
Diagnosis, No. (%), n		
Hyper tension (1^*^)	13 (52.0)	11 (44.0)
Heart disease (1^*^)	2 (8.0)	0 (0)
Diabetes Mellitus	5 (20.0)	3 (12.0)
Medication, 3 and over	10 (40.0)	11 (44.0)
Blood test, mean ± SD		
Total cholesterol, mg/dL	212.6 ± 36.9	202.8 ± 32.2
Triglyceride, mg/dL	146.8 ± 73.7	130.4 ± 112.3
Glucose, mg/dL	116.3 ± 27.1	110.4 ± 23.4
HA1c, %	5.6 ± 0.6	5.4 ± 0.5
Cognitive functions, mean ± SD		
MMSE, score	26.8 ± 1.8	26.6 ± 1.6
WMS-LM I, score	12.5 ± 5.9	12.0 ± 4.9
WMS-LM II, score	8.2 ± 5.4	6.9 ± 5.0
DSC, s	47.5 ± 15.4	44.3 ± 16.3
LVFT, score	16.0 ± 5.3	16.9 ± 6.0
CVFT, score	33.1 ± 6.9	31.2 ± 7.7
SCWT-I, s	22.6 ± 9.7	23.4 ± 11.1
SCWT-III, s	42.0 ± 13.7	41.5 ± 17.7
TMIG index, mean ± SD, score		
IADL	5.0 ± 0.2	4.9 ± 0.3
Intellectual activity	3.8 ± 0.4	3.8 ± 0.4
Social role	3.6 ± 0.9	3.6 ± 0.8
Total	12.3 ± 1.1	12.3 ± 0.9
GDS, mean ± SD, score	3.0 ± 2.1	2.6 ± 2.0

SD; standard deviation, MMSE; mini-mental state examination, WMS-LM II; Logical Memory II subtest of the Wechsler memory scale-revised, LVFT; letter verbal fluency test, CVFT; category verbal fluency test, DSC; digit-symbol cording, SCWT; Stroop Color and Word Test, TMIG index; Tokyo Metropolitan Institute of Gerontology index, GDS; Geriatric Depression Scale. ^*^ missing value.

### Changes in cognitive function

**Figure 2:**
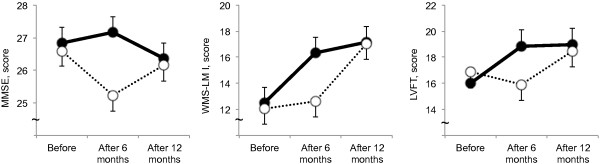
**Changes in the MMSE, WMS, and LVFT scores.** MMSE; mini-mental state examination, WMS-LM I; Logical Memory I subtest of the Wechsler memory scale-revised, LVFT; letter verbal fluency test. Panels showed change in MMSE, WMS-LM I, and LVFT scores before, after 6 months, and after 12 months intervention. Solid and dashed lines indicate the exercise and control groups, respectively. Group mean and standard errors are shown in older adults with amnestic mild cognitive impairment. The linear mixed models revealed significant group × time interactions in MMSE (*P* = 0.04), WMS-LM I (*P* = 0.03), and LVFT (*P* = 0.02).

Table 2 shows changes in cognitive scores over the 12 months across the groups. On the MMSE, there was a group × time interaction (*P* = 0.04) indicated benefit of the exercise over time (Figure 2). Although there were no main effects of group or time, the control group showed significant decline in the MMSE score after 6 months compared to before intervention (*P* < 0.05) and there was significant differences between the groups at after 6 months (*P* < 0.05).

**Table 2:** Comparison of cognitive functions between exercise and control groups

	**Mean difference (95% CI) between before and after 6 months**		**Mean difference (95% CI) between before and after 12 months**		**Time**			**Group**			**Group × time**		
	**Exercise (n=25)**	**Control (n=25)**	**Exercise (n=25)**	**Control (n=25)**	**df**	**F value**	**P value**	**df**	**F value**	**P value**	**df**	**F value**	**P value**
MMSE	0.32 (−0.96−1.60)	−1.37 (−2.66−−0.07)	−0.47 (−1.75−0.81)	−0.44 (−1.74−0.86)	92.1	1.17	0.32 ^d^	48.0	2.2	0.14 ^g^	92.1	3.4	0.04
WMS-LM I	3.83 (1.40−6.25)	0.60 (−1.87−3.06)	4.62 (2.19−7.05)	5.00 (2.53−7.46)	90.9	23.0	<0.01 ^a, b, e, f^	47.8	0.9	0.34 ^g^	90.9	3.9	0.03
WMS-LM II	3.79 (1.49−6.10)	2.09 (−0.25−4.43)	5.13 (2.82−7.43)	6.20 (3.86−8.54)	91.1	35.4	<0.01 ^a, b, e, f^	48.1	1.1	0.30	91.1	2.1	0.13
DSC	−0.19 (−4.20−3.81)	3.27 (−0.80−7.34)	3.64 (−0.36−7.64)	3.73 (−0.34−7.80)	90.4	5.0	<0.01	48.0	0.2	0.65	90.4	1.4	0.25
LVFT	2.87 (0.57−5.17)	−0.97 (−3.31−1.37)	2.99 (0.69−5.30)	1.61 (−0.73−3.95)	91.2	5.9	<0.01	48.3	0.3	0.59	91.2	4.1	0.02
CVFT	1.54 (−1.27−4.35)	−1.81 (−4.67−1.04)	1.33 (−1.48−4.14)	−1.61 (−4.46−1.25)	91.2	0.02	0.98	48.5	3.1	0.08	91.2	2.5	0.09
SCWT-I	−3.30 (−7.56−0.96)	−0.98 (−5.30−3.34)	−3.68 (−7.94−0.58)	−3.20 (−7.52−1.12)	91.5	3.9	0.02	47.7	0.73	0.40	91.5	0.5	0.62
SCWT-III	−4.70 (−12.72−3.33)	0.15 (−8.00−8.30)	−2.61 (−10.64−5.42)	−4.57 (−12.72−3.58)	92.0	1.2	0.31	48.8	0.009	0.92	92.0	1.1	0.34

MMSE; mini-mental state examination, WMS-LM; Logical Memory subtest of the Wechsler memory scale-revised, DSC; digit-symbol cording, LVFT; letter verbal fluency test, CVFT; category verbal fluency test, SCWT; Stroop Color and Word Test, TMIG; Tokyo Metropolitan Institute of Gerontology index.

^a^ P < .05; significant differences between before and after 6 months in the exercise group, ^b^ P < .05; significant differences between before and after 12 months in the exercise group, ^c^ P < .05; significant differences between after 6 months and 12 months in the exercise group, ^d^ P < .05; significant differences between before and after 6 months in the control group, ^e^ P < .05; significant differences between before and after 12 months in the control group, ^f^ P < .05; significant differences between after 6 months and 12 months in the control group, ^g^ P < .05; significant differences between the exercise and control groups at after 6 months, ^h^ P < .01; significant differences between the exercise and control groups at after 12 months.

For the WMS-LM I, there was a group × time interaction (*P* = 0.03); there were an overall effect of time and no main effect of group (Figure 2). In the post hoc analysis, the exercise group showed significant increase in the WMS-LM I score after 6 and 12 months compared to before intervention (*P* < 0.05) and the control group showed significant increase after 12 months compared to before intervention and after 6 months (*P* < 0.05). There was a significant difference between the groups at 6 months (*P* < 0.05).

On the LVFT, there was a group × time interaction (*P* = 0.02); there were an overall effect of time and no main effect of group (Figure 2). In the post hoc analysis, There were no significant differences between the times and groups.

The WMS-LM II, DSC, and SCWT-I showed main effect of time, although there were no group × time interaction and main effect of group. In the post hoc analysis, the exercise group showed significant increase in the WMS-LM II score after 6 and 12 months compared to before intervention (*P* < 0.05) and the control group showed significant increase after 12 months compared to before intervention and after 6 months (*P* < 0.05). There were no significant differences between the groups at each timepoints (Table 2).

## Discussion

There was a significant group × time interaction on the MMSE, WMS-LM I, and LVFT scores. Twelve months of multicomponent exercise improved cognitive function in older adults with aMCI relative to the education control group. In particular, positive effects were observed for general cognitive function, immediate memory, and language ability, which is consistent with findings in cognitively intact adults [28]. A recent randomized controlled trial has been described as providing verification of the benefits of exercise in elderly adults with MCI [23]. In that study, 152 participants were randomly assigned to an aerobic exercise group and a non-aerobic exercise group, and to a vitamin B group and a placebo group, and a one-year intervention was carried out. The participants exercised twice weekly for 60 minutes each time. For the aerobic exercises, they walked together in groups. The results showed that aerobic exercise has no significant effect in improving cognitive function. However, these results were based on an intention-to-treat analysis, which included 30 participants who did not attend the exercise sessions. Had those elderly adults who had a high attendance rate among the aerobic exercise group been included in the analysis, then the results would have shown increased memory and attention, confirming the effectiveness of aerobic exercise in elder adults with MCI, though only to a limited extent. In another recent report, when elderly adults with MCI (a mean age of 70 years) engaged in aerobic exercise four times every week over the course of six months with a heart rate reserve of 75% to 85%, executive function significantly improved [16].

The present study shows that significant interactions were observed in general cognitive function, immediate memory, and verbal fluency between the groups, although intervention effects on delayed memory, processing speed, and executive control did not reach significance. Lautenschlager et al. reported that physical activity and behavioral interventions improve general cognitive function [19]. The multicomponent exercise training used in the current study also included aerobic exercise and behavioral interventions, such as self-monitoring of home-based exercise. Our results further supported the idea that a composite approach including aerobic exercise and behavioral interventions can have beneficial effects on cognitive function in aMCI patients.

Older adults with aMCI exhibit greater decreases in memory function than in other cognitive functions, relative to healthy older adults [40]. The cognitive deficits in aMCI increase the risk of conversion from MCI to AD [11,12]. Enhancing cognitive function, especially memory, in MCI may prevent conversion from MCI to AD in older adults. Our multicomponent exercise program involved cognitive loads during exercise. In other words, exercise was conducted under multitask conditions such as dual-task stimulation or while learning tasks during the exercises [41]. Our multicomponent exercise program, involving aerobic exercise, muscle strength, and additional cognitive demand, has some advantages for improving cognitive function over aerobic exercise alone, including possibly increasing logical memory in older adults with aMCI. The WMS-LM I scores in the education control group increased significantly at 12 months compared to before and after 6 months. The education control group received reports of the results of the three assessments and lectures regarding health. We suggest that these educational approaches may be useful in maintaining healthy behavior, such as starting cognitive training or intellectual activities. In fact, the subjects in the control group had fewer cessations of intellectual activity, e.g. culture lessons, than the exercise group during the 12-month period (−9% vs. −19%).

Baker et al. reported that high intensity aerobic exercise increased VFT scores in older women with MCI [16]. Early in the dementia process, the ability to consciously access lexical information about a target word is impaired while the overall semantic system is intact [42], whereas later in the disease, the integrity of the entire system is compromised, resulting in impaired name recall in structured tasks and spontaneous conversation [42,43]. Fluency tests tap into lexical and semantic retrieval operations and may be able to measure these specific aspects of language breakdown in aMCI patients. In a functional neuroimaging study using near infrared spectroscopy, patients with AD showed decreased brain activation patterns compared with healthy controls during the conduct of VFT. Significant correlations between brain activation and performance in the LVFT for dementia patients were found [44]. In the present study, multicomponent exercise provided positive effects on LVFT scores in the aMCI subjects, who had a higher risk of dementia [45].

The present study has several limitations. The small sample size means that replication with a larger group of adults with MCI would be beneficial. Other limitations include unknown group differences in the risk factors of cognitive decline and AD, such as apolipoprotein E ε4 genotypes [46], and inflammation [47], although there were no significant differences between the groups in hypertension, diabetes mellitus, medications, biomarkers of lipid metabolism, physical performance, instrumental ADL functioning, and depressive moods. In addition, it is possible that the improvement in the exercise group resulted from the social contact that the intervention group received. This possibility cannot be completely excluded with the present design and should be addressed in future studies.

## Conclusions

Twelve months of exercise improved cognitive function in older adults with aMCI relative to the education control group. In particular, positive effects were observed for general cognitive function, immediate memory, and language ability. A future follow-up investigation is required to determine whether the effect is associated with prevention or delayed onset of dementia in older adults with aMCI.

## Abbreviations

aMCI: Amnestic mild cognitive impairment; AD: Alzheimer’s dementia; CDR: Clinical dementia rating; WMS-LM: Wechsler memory scale-logical memory; ADL: Activities of daily living; CONSORT: Consolidated standards of reporting trials; MMSE: Mini-mental state examination; DSC: Digit symbol-coding; LVFT: Letter verbal fluency test; CVFT: Category verbal fluency test; SCWT: Stroop color and word test; ITT: Intention-to-treat.

## Competing interests

The authors declare that they have no competing interests.

## Authors' contributions

Conception of the idea for the study: TS and HS. Development of the protocol and organization: TS, HS, HM, TD, and DY. Acquisition of participants, study management, and statistical analysis: HS, HM, TD, DY, KT, YA, KU, SL, and HP. All authors contributed to the interpretation of the data and drafting the article and provided final approval of the version to be published. All authors read and approved the final manuscript.

## Pre-publication history

The pre-publication history for this paper can be accessed here:

http://www.biomedcentral.com/1471-2377/12/128/prepub
